# Effects of neonatal stress on gamma oscillations in hippocampus

**DOI:** 10.1038/srep29007

**Published:** 2016-07-01

**Authors:** Sally Dricks

**Affiliations:** 1 Department of Physiology and Pharmacology, SUNY Downstate Medical Center, Brooklyn, NY 11203 USA.

## Abstract

Chronic early life stress increases adult risk for depression, bipolar disorder and schizophrenia, illnesses characterized by aberrant functions of cognition and memory. We asked whether chronic early life stress disrupts maturation of gamma oscillations, on which these functions depend. Lifelong impairment of the stress response results from separation of rat pups from the dam for three hours per day during a critical period of hippocampal development (PNDs 2–14). Parvalbumin-expressing interneurons, including the basket cell network which is fundamental to gamma oscillations, are reduced in number in post mortem studies of bipolar disorder and schizophrenia, and in chronically-stressed adult rats. To determine effects of chronic early life stress on gamma oscillations, we separated pups from dams once each day on PNDs 2–14 and recorded *in vitro* at PNDs 15–21. In control pups, separated for 15 minutes per day, gamma power had highly significant correlations with both age (p = 0.0022) and weight (p = 0.0024); gamma in pups separated for 180 minutes per day was not correlated with either factor. ANCOVA indicated significant differences between the groups in both measures. These findings indicate that chronic early life stress can disrupt maturation of the gamma oscillation network.

Chronic early-life stress has been shown in both animal and human studies to have a profound and lifelong impact on physical and psychological health, as correlated with increased rates of depression, schizophrenia, bipolar and anxiety-related disorders, alcohol and drug abuse and suicide, as well as cognitive and memory-related impairments^1^,^2^. Early-life adversity has been shown to impair in a virtually irreversible manner the negative-feedback system that normally returns circulating corticosteroids to basal levels in the wake of a stressor; the result is neurotoxic levels of exposure to corticosteroids beginning in the developing nervous system and continuing throughout the lifetime.

A well-established rearing protocol known as HMS180, which models chronic early-life adversity, has shown that separation of rat pups from their dam for three hours per day during a critical period of brain development, postnatal days 2 through 14, induces lifelong impairment of the stress response as a result of premature and excessive exposure to corticosteroids, with physiological and behavioral effects resembling those seen as a result of early-life trauma, neglect and abuse in human subjects^3^.

Postmortem studies of subjects with schizophrenia and bipolar disorder^4^,^5^, as well as studies of rats subjected to chronic stress in adulthood^6^,^7^, show significantly reduced numbers of parvalbumin-immunoreactive interneurons relative to controls. Approximately 60% of the parvalbumin-immunoreactive interneuron population in hippocampus is comprised of basket cells, interneurons which provide powerful perisomatic inhibition to pyramidal and granule cells; the rapid, synchronous firing of parvalbumin-expressing basket cells induces the rhythmic inhibitory postsynaptic potentials fundamental to gamma oscillations^8^,^9^. Gamma oscillations are intrinsic to cognition, perception and memory^10^,^11^ and aberrant in patients with schizophrenia and bipolar disorder^12^,^13^, illnesses thought to be linked to neurodevelopmental sequela in those genetically predisposed^14^,^15^, and for which risk is greatly increased by a history of chronic neglect, abuse or trauma in early life^1^.

In rat pups, PNDs 2 through 14 comprise the “stress hypo-response period” (SHRP), during which the stress response is, in effect, “offline”: under any less than extreme conditions, during the SHRP pups will mount little or no hormonal response to such aversive stimuli as surgery, handling, electric shock or extremes of temperature^16^. The dramatic increases in levels of adrenocorticotropin hormone and corticosterone that occur in response to a stressor before and after this phase are absent, and basal levels of circulating corticosterone fall markedly. Sapolsky and Meaney^16^ proposed that the SHRP is an evolutionary adaptation that serves to protect the nascent central nervous system from the catabolic effects on neural development of excessive exposure to glucocorticoids.

Sufficiently severe stress, however, such as that produced by a single 24-hour separation from the dam, can “break through” the SHRP to cause a significant rise in plasma corticosteroids. The negative-feedback system that turns off the release of glucocorticoids in the healthy adult is not fully functional until puberty^17^ so any exposure to glucocorticoids during the SHRP is extended, and the maturing feedback function commensurately impaired. While PVBCs in adult animals are known to be adversely affected by chronic stress, these cells may be especially vulnerable to developmental insult during the SHRP via chronic exposure to elevated levels of glucocorticoids, as well as during the protracted period of development that follows, given that they are still morphologically and electrophysiologically immature until PND25^18^.

Development of the neuroendocrine stress response is largely under maternal control in the rat: frequent licking and grooming of neonates by the dam is necessary for proper maturation of the hypothalamic-pituitary-adrenal axis. Chronic stress induced by low levels of licking and grooming during the critical period of HPA development induces epigenetic changes at a promoter region of the glucocorticoid receptor gene on or around postnatal day 8, resulting in the permanent down-regulation of glucocorticoid receptor expression in hippocampus^19^,^20^. As a result, the negative fast feedback signal is progressively degraded, so that following activation of the stress axis, glucocorticoids continue to be released for an extended time. Even after short-term stress, both adult and juvenile animals show altered dendritic morphology in pyramidal cells of the hippocampus and medial prefrontal cortex^21^, atrophy of pyramidal apical dendrites in hippocampus^22^ and decreased expression of synaptic spines^23^. Subsequently, lifelong exposure to elevated levels of glucocorticoids has been shown to have increasingly profound neurotoxic effects, including acceleration of normal age-related reduction of the glucocorticoid receptor population in hippocampus^24^, impairment of adult neurogenesis^25^, and acceleration of age-related loss of hippocampal volume due to dendritic atrophy and remodeling^26^.

Characteristically fast- spiking and non-accommodating, parvalbumin-expressing basket cells (PVBCs) form dense axonal arbors exclusively around the soma and proximal dendrites of pyramidal and granule cells, largely determining their output. Facilitating synchronization of PVBCs is that as a population they are networked via both chemical synapses and gap junctions. Widely thought to be the “timekeepers” of cortical network oscillations^8^, their precisely synchronized firing induces the inhibitory post-synaptic currents from pyramidal cells which constitute the field potential in cholinergically-induced gamma oscillations^27^.

While development of hippocampal pyramidal cells is largely complete at PND21, PVBCs require four to five postnatal weeks to become fully mature in their biophysical properties^18^. As the morphology, connectivity and intrinsic firing properties of PVBCs develop from slow- to fast-spiking, gamma oscillations increase in amplitude and frequency^28^. Duration of the action potential decreases by nearly half between PND5 and PND25, as does the decay constant of inhibitory post-synaptic currents, while the threshold for initiation of firing and maximal firing frequency increase. At maturity, each PVBC contacts about 60 other PVBCs and about 1,500 granule or pyramidal cells^29^.

Gamma oscillations have been recorded and described *in vitro* and *in vivo* in rodents of all ages, in a number of brain structures. However, the progressive maturation of hippocampal gamma oscillations, approximately spanning postnatal days 12 to 25 in rat pups, which occurs in parallel with and as a function of the morphological and electrophysiologcal maturation of PVBCs^18^, has not to our knowledge been documented with data points taken at time frames appropriate to the rapid changes occurring during this period, either *in vivo* or *in vitro. In vitro* studies of hippocampal gamma oscillations have in fact typically pooled data from animals between 14 and 22 days old^27^,^30^, in part, at least, because of the relative ease and consistency with which oscillations can be induced and maintained in the acute slice preparation during that phase, although *in vivo* studies using awake behaving, mature animals demonstrate that gamma oscillations continue to occur in the adult. The practice of pooling of data from animals which are still morphologically and electrophysiologically immature assumes that there are no significant age-related differences between 14- and 22-day old pups or between those pups and adults which would need to be considered in interpreting experimental results. This assumption is in conflict with what we now know about the electrophysiological maturation of cellular networks in hippocampus.

Impairment of parvalbumin-expressing interneuron networks in hippocampus or prefrontal cortex, by such mechanisms as increased asynchronous GABA release or loss of NMDA receptors^31^, disrupts gamma oscillations. Aberrant network activity caused by impairment of PVBC activity is widely thought to underlie the disturbances in mood, cognition and memory associated with mental illnesses^32^,^33^. PV+ interneurons have been shown to be reduced in prefrontal cortex and hippocampus in postmortem studies of human patients diagnosed with schizophrenia, major depression and bipolar disorder^4^,^5^, illnesses in which abnormal gamma oscillations are also observed^34^,^35^.

In linking impairment of PVBC networks in hippocampus and neocortex to psychiatric illness, questions still remain as to whether changes in this interneuron population precede the onset of psychiatric illness, or are a result of psychiatric illness or psychotropic medications, or are possibly a direct etiological factor in illness development. Studies of postmortem tissue^14^,^15^ indicated that the extent of reduction in the number of parvalbumin-immunoreactive cells in schizophrenia subjects was not correlated with age of the subject, duration or severity of their illness, their antipsychotic drug treatment history or incidence of drug abuse. These findings imply that the impairment of PVBC network function does not progress with the course or severity of the illness and is not a consequence of the use of psychotropic substances. These authors and others have proposed a neurodevelopmental origin for the deficit in function of parvalbumin-expressing interneurons, noting the relatively late postnatal onset of parvalbumin expression in contrast to other major neuronal calcium-binding proteins and that the parvalbumin-expressing interneuron population is the last to mature.

Given the sensitivity to perturbation of neuronal development during the SHRP, we asked whether gamma oscillations in rat pups would be affected by repeated episodes of maternal separation and concomitant premature exposure to glucocorticoids, and if so, whether that effect would be mediated by compromised development of the PVBC network.

Slice electrophysiology was utilized for this study because the slice preparation provides very tightly controllable and reproducible conditions ideal for this type of study; such variables as recording temperature and composition of the extracellular milieu are uniform for each experiment. Especially important to our study is that input to the slice is limited to that which is administered in the same manner each time in the form of pharmacological or electrophysiological manipulation, criteria impossible to achieve with *in vivo* designs. Isolated from the oscillatory activity of other brain regions, the variables affecting gamma activity can be defined in a simplified paradigm, and modified as needed. For these reasons, slice preparations have been very important in studies of the instrumental role of perisomatic inhibition by PVBCs in neuronal oscillations^36^,^37^,^38^,^39^.

Carbachol, an agonist of both muscarinic and nicotinic acetylcholinergic receptors, was chosen to induce gamma oscillations because it specifically mimics *in vivo* acetylcholine release from the dense cholinergic projection to the hippocampus from the medial septum/diagonal band of Broca^40^,^41^. In addition, carbachol produces oscillations having several of the most important features of gamma oscillations *in vivo*, including low frequency pyramidal cell firing which is phase-locked to the oscillation^36^. Carbachol has been preferentially employed to induce hippocampal gamma oscillations *in vitro* by many prominent investigators in this field^27^,^30^, including those whose methods have been cited in the present work, facilitating comparison of data from numerous labs.

As previously described^42^, each litter of pups was exposed to one of two rearing conditions from PNDs 2 through 14, inclusive: handling with separation from the dam for 15 minutes per day (HMS15) or handling with separation for 180 minutes per day (HMS180). After PND14, pups and dams in both groups were undisturbed except for standard institutional animal facility handling. *In vitro* electrophysiological studies were conducted on PNDs 15 through 21 on acute brain sections of dorsal hippocampus, using bath-applied carbachol (12.5 μM), a cholinergic receptor agonist, to induce gamma oscillations (Figs 1 and 2). Recordings were made from the pyramidal cell layer of CA3, distal to the fornix. Immunohistological staining of hippocampal tissue for parvalbumin (Fig. 3) was performed at the same longitudinal level of hippocampus.

## Methods and Experimental Design

All experiments were approved by the Animal Care and Use Committee at SUNY Downstate Medical Center and were in compliance with the guidelines of the institutional ethical code.

### Culling and rearing of animals

Each group of pups was born in-house from three timed-pregnant Sprague-Dawley dams (Charles River, Kingston, NY). Dams were housed individually in adjacent polycarbonate cages with ad libitum access to food and water on a 12 hours: 12 hours light: dark cycle (lights on at 7:00 AM) and gave birth within close temporal proximity. The day of birth is referred to here as Postnatal Day 0 (PND 0). On PND1, 10–12 males and two females were placed with an unrelated dam; females were included in the litter to avoid single-sex litter effects^36^.

As previously described^42^, each foster litter was exposed to one of two rearing conditions from PNDs 2 through 14, inclusive: handling with separation from the dam for 15 minutes per day (HMS15) or handling with separation for 180 minutes per day (HMS180). For separation periods, pups were moved to an adjacent room and placed in a temperature- and humidity-controlled incubator (Lyon Technologies/Marsh Farms, Chula Vista, CA, Animal Intensive Care Unit with Digital Display). Separations were done at varying times during the light cycle (typically between 11am and 4pm) in order to increase stress on both dams and pups by limiting predictability. Other than the duration of separations from the dam, both HMS groups were treated in the same manner. After PND14, pups and dams in both groups were undisturbed except for standard institutional animal facility handling.

### Electrophysiology

HMS-reared males aged 15–21 days were anesthetized with evaporating isoflurane and decapitated with a guillotine. The brain was quickly removed and submerged in ice-cold cutting solution (~4 °C) containing the following (in mM): sucrose, 252; KCl, 3; NaHCO_3_, 26; CaCl_2_, 1; MgSO_4_, 5; NaH_2_PO_4_, 1.25; glucose, 10, prepared with ultra pure water and bubbled with 95% O_2_/5% CO_2_ (carbogen gas)^36^. The brain was halved through the midline for use in electrophysiology and immunohistochemistry.

For electrophysiology studies, 400-μM thick slices of dorsal hippocampus were made using a Vibratome Series 3000 Plus (Ted Pella, Redding, CA). Slices were placed for ≥60 minutes on nylon netting submerged in room temperature extracellular solution of the following composition (in mM): NaCl, 126; KCl, 3; NaHCO_3_, 26; CaCl_2_, 1; MgSO_4_, 5; NaH_2_PO_4_, 1.25; glucose, 10, prepared with ultra pure water and bubbled with 95% O_2_/5% CO_2_ (carbogen gas)^36^.

Electrophysiology experiments were conducted using an Axon Multiclamp 700 A amplifier and an Axon Digidata 1332 A Digitizer (both, Molecular Devices, Sunnyvale, CA). Axon pClamp version 9.2 software (Molecular Devices) was used for data acquisition. Input was low pass filtered at 2 kHz and high pass filtered at 0.10 kHz prior to digitization, and sampled online at 6.667 kHz.

Slices were visualized using a Nikon Eclipse E600FN upright compound microscope with a 4X objective, Cool Snap ES2 High-Performance Interline CCD Camera and Nikon NIS Elements image acquisition software. Oscillations were visualized in real time on a spectrum analyzer using a DSO-2100 digital storage oscilloscope and software (Link Instruments, Fairfield, NJ) on a Gateway Solo 1400 notebook computer.

Recordings were made in a commercial submerged-type slice chamber (Warner Instruments Model RL-27L, Warner Instruments, Hamden, CT) using a Burleigh Piezoelectric micromanipulator (Thorlabs, Newton, NJ). Solution was maintained at 29.8–31.5 °C by an inline heater (Warner TC324B Temperature Controller, Warner Instruments, Hamden, CT), with a gravity-driven extracellular solution flow rate of 3.5–5.5 mL/min^36^.

Recording electrodes (3.5–5 MΩ) were pulled from thin-walled borosilicate glass capillaries, filled with the extracellular solution described above^36^ and placed in dorsal CA3, in the pyramidal cell layer and distal to the fimbria. Baseline recordings of local field potentials, three to seven minutes in duration, were made prior to addition of 12.5 μM carbachol (carbamoylcholine chloride) to the extracellular solution supply. Oscillations continued for as long as recording continued, which on some occasions exceeded three hours; oscillations could be rapidly terminated by 1 μM atropine, a competitive muscarinic receptor antagonist.

All chemicals and drugs for electrophysiology were purchased from Sigma-Aldrich except magnesium sulfate hydrate, purchased from Fluka Biochemika (Buchs, Switzerland), and sucrose, purchased from Fisher Scientific.

### Data collection

Electrophysiological recordings were analyzed off-line using custom scripts with commercial software (Matlab 7.10, The MathWorks Inc., Natick, MA, 2000). In order to exclude harmonics of the gamma oscillations from power analysis, the bandwidth was filtered offline to a range of 20 to 40 Hz. Fundamental frequencies recorded during all experiments were within the 20 to 40 Hz band width prior to filtering. Analyses of gamma oscillations were in all cases performed on the 20-minute interval between 30 minutes and 50 minutes from the start of the recording. One brain slice was analyzed from each rat pup.

The gamma power data of one HMS180 pup was not used because it was 5.5 standard deviations above the mean of all HMS180 pups recorded in this study and 6 standard deviations above the mean of all HMS15 pups recorded in this study, exceeding our criteria of ≥2.5 standard deviations for determination of an outlier. Gamma oscillation frequency data from this pup, although within criteria, was also excluded as representative of the same recording. Naive rats of similar age and under the similar experimental conditions have produced oscillations of comparable gamma power in our lab (unpublished data) so we do not believe that investigator or equipment error accounted for this result.

### Immunohistochemistry

The remaining half of the brain was submerged in a solution of paraformaldehyde (4% in PBS, Boston BioProducts, Ashland, MA) at 4 °C for 24 to 48 hours, stored in phosphate-buffered saline (PBS) at 4 °C, then sliced horizontally at a 40-μM thickness on a Leica VT 1200S Vibratome (Leica Microsystems, Buffalo Grove, IL). Sections used for immunohistochemistry were from the same longitudinal level of the hippocampus as those used for electrophysiology.

Sections were stored in PBS at 4 °C, washed three times for five minutes in fresh PBS, incubated for 60 minutes in 1% H_2_O_2_ in PBS, then washed three times for five minutes in PBS with 0.05% Tween-20 (PBST) (Acros Organics, Geel, Belgium). Sections were incubated for 60 minutes in 3% normal donkey serum (Jackson ImmunoResearch, West Grove, PA) and 0.5% Triton X-100 (Acros Organics, Geel, Belgium) in PBS, then for one hour at 4 °C with a monoclonal mouse anti-parvalbumin antibody specific to the calcium-bound form (Millipore MAB1572, dilution 1:3000) diluted in 3% normal donkey serum and 0.5% Triton X-100 in PBS. Sections were washed with PBST four times for five minutes, then incubated at room temperature for two hours with a biotinylated, rat-adsorbed horse anti-mouse IgG antibody (Abcam BA2001, dilution 1:100) diluted in 3% normal goat serum and 0.5% Triton X-100 in PBS and washed with PBST four times for five minutes.

Sections were then incubated with an avidin-biotin enzyme complex (ABC Vectastain Elite Standard Kit, Vector Laboratories, Burlingame, CA) for one hour and washed with PBST four times for five minutes each. DAB (3,3′-diaminobenzidine) (DAB-Plus Reagent Set, Novex, Life Sciences/Invitrogen, Grand Island, NY) was prepared with distilled H_2_O according to manufacturers’ directions, briefly centrifuged and pushed through a 22-μm filter by syringe. Sections were then incubated in filtered DAB for three to five minutes, washed in PBST and PBS three times for five minutes and then in distilled H_2_O for five minutes before being immersed in PBST for mounting on charged glass slides (Fisherbrand Superfrost Plus Microscope slides). ProLong Gold Antifade Reagent (Molecular Probes/Life Technologies) was applied to sections before coverslips were placed.

Whole-slide imaging was performed by New York University’s Office of Collaborative Science Experimental Pathology Histology Core Lab (http://ocs.med.nyu.edu/histopathology/contact-us) on a Leica SCN400F scanner at 40X magnification at a resolution of 0.250 microns per pixel. Native format image files were re-saved for use with ImageJ in tiff format with Aperio ImageScope v12.1.0.5029 (Leica Microsystems Inc., Buffalo Grove, IL).

### Data collection

Boundaries of hippocampal subfields were defined on each section using guides from van Strien 2009^43^. The Cell Counter plug-in feature in ImageJ was used to mark and tally cells manually identified by the investigator.

Data from each subfield of sections taken from the same animal were averaged. SEMs were calculated as the standard error of the mean of sections from the same pup. “Total PV+ cells” is the number of parvalbumin-labeled cells per hippocampal section, averaged over multiple sections from the same pup.

Immunohistological data was analyzed using Matlab 7.10 and ImageJ 1.49 g (Rasband, W.S., ImageJ, U.S. National Institutes of Health, Bethesda, Maryland, http://imagej.nih.gov/ij/, 1997–2012).

### Statistical analysis

Data was normally distributed. Comparison of statistical models for analysis of HMS15 data indicated that it best fit a linear model, except for analyses involving gamma power, which best fit a semi-log model, with the X axis on a linear scale and the Y axis (gamma power) on a log scale. For this reason, gamma power data for both groups was transformed by using the square root of gamma power for all figures and analyses, by which both figures and analyses preferentially fit linear models. Parametric statistical analyses utilizing linear regression methods (Pearson correlation, multiple regression analysis and ANCOVA) were therefore employed throughout.

Figures were produced using Aperio ImageScope v12.1.0.5029, Matlab 7.10, Adobe Photoshop and Adobe Illustrator software versions CS5.5 and CC 2015.

## Results

### Maturation of gamma as predicted by age and weight

**Figure 4:**
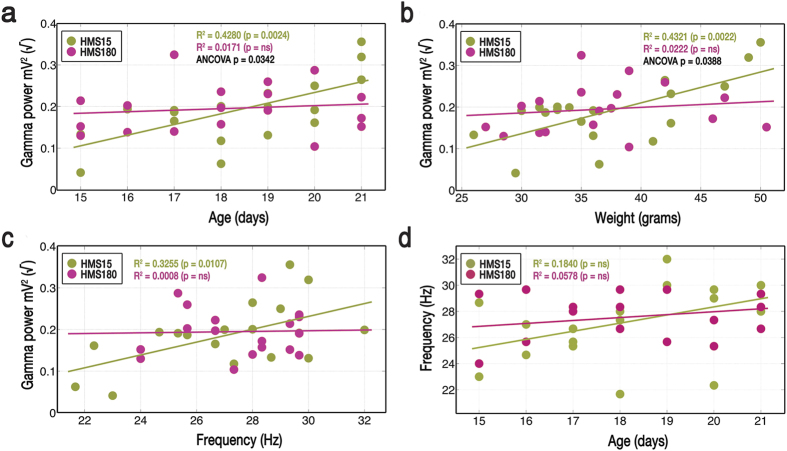
Correlations between measures of development and gamma oscillation power or frequency differ by rearing condition (brief vs. extended maternal separations). Cholinergic-receptor agonist carbachol (12.5 μM) was bath-applied to induce oscillations in 400 μM-thick slices, which were recorded from the pyramidal cell layer of CA3 in dorsal hippocampus. Gamma power was transformed by using the square root of gamma power in both figures and analyses, by which both were made linear. (**a**) Gamma power varied with age in HMS15 pups; the correlation was much stronger between PNDs 19–21 than between PNDs 15–18. In HMS180 pups, no correlation was seen between age and gamma power overall or within either time frame. ANCOVA indicated that respective relationships between age and gamma power differed between the two HMS groups. (**b**) Gamma power varied with weight in HMS15 pups, with the correlation much stronger at >36 grams than at ≤36 grams. In HMS180 pups no correlation was seen between weight and gamma power overall or within either weight range. ANCOVA indicated that respective relationships between weight and gamma power differed between the two HMS groups. (**c**) Gamma oscillation frequency varied with gamma power in HMS15 pups; no correlation was seen between these factors in HMS180 pups. (**d**) Correlations between age and gamma oscillation frequency were not observed in either HMS group.

In agreement with previous studies of the electrophysiological maturation of fast-spiking interneurons in hippocampus in the neonate^18^, the power of gamma oscillations increased with age in the brief maternal separation (HMS15) group, with a highly significant coefficient of determination between those factors (R^2^ = 0.4280, p = 0.0024, n = 19) (Fig. 4a). In contrast, for the extended maternal separation (HMS180) pups, age would explain less than 2% of the variance in gamma power (R^2^ = 0.0171, p = 0.6049, n = 18). Analysis of covariance (ANCOVA) showed that the relationships between age and gamma power were significantly different in HMS15 versus HMS180 pups (p = 0.0342).

The strength of the correlation between age and gamma power increased markedly across time in HMS15 pups. Between PNDs 15 and 18, the magnitude of gamma power in both HMS15 and HMS180 pups was not related to the factor of age (HMS15: R^2^ = 0.0198, p = 0.6996, n = 10; HMS180: R^2^ = 0.0880, p = 0.4045, n = 10). However, between PNDs 19 and 21, age predicted over 50% of the variance in gamma power in HMS15 pups (R^2^ = 0.5630, p = 0.0199, n = 9). ANCOVA showed that in HMS15 pups, the correlation between age and gamma power between postnatal days 15 and 18 differed significantly from that seen between postnatal days 19 and 21 (p = 0.0112). For HMS180 pups, the relationship between age and gamma power at PNDs 19 to 21 was relatively unchanged (R^2^ = 0.1209, p = 0.3979, n = 8).

Weight was a predictor of change in gamma power for HMS15 pups, explaining over 40% of the variance (R^2^ = 0.4321, p = 0.0022, n = 19) (Fig. 4b), in contrast to HMS180 animals, R^2^ = 0.0222 (p = 0.5553, n = 18), where no correlation was observed. ANCOVA indicated that HMS15 and HMS180 pups differed significantly in their respective correlations between weight and gamma power (p = 0.0388).

The strength of the correlation between weight and gamma power changed in both groups as they increased in size. At or below the median weight of 36 grams, the groups had similarly nominal and non-significant relationships between weight and gamma power (HMS15: R^2^ = 0.1285, p = 0.2797, n = 9; HMS180: R^2^ = 0.2604, p = 0.1604, n = 9). But in HMS15 pups weighing >36 grams, weight was a very strong and highly significant predictor of over 80% of gamma power variance (R^2^ = 0.8201, p = 0.0019, n = 10). ANCOVA showed that in HMS15 pups, the correlation between weight and gamma power between pups weighing ≤36 grams differed significantly from that in pups weighing >36 grams (p = 0.0388). For HMS180 pups >36 grams, the correlation between weight and gamma power (R^2^ = 0.0388, p = 0.6125, n = 9) was notably weaker than for pups at ≤36 grams.

HMS15 pups showed a significant correlation between gamma oscillation frequency and gamma power (R^2^ = 0.3255, p = 0.0107, n = 19) (Fig. 4c). In contrast, HMS180 pups showed virtually no relationship between those factors (R^2^ = 0.0008, p = 0.9125, n = 18).

Multiple regression analysis revealed a highly significant interaction between the factors of weight and gamma oscillation frequency in predicting gamma power in HMS15 pups, such that over 60% of the variance in gamma power could be explained by these two factors (multiple R^2^ = 0.6154, p = 0.0003, n = 19). Strong and significant interaction between age and oscillation frequency accounted for over 40% of gamma variance in HMS15 pups (multiple R^2^ = 0.4315, p = 0.0055, n = 19). In contrast, interaction between weight and oscillation frequency did not predict gamma power variance in HMS180 pups (multiple R^2^ = 0.0175, p = 0.8705, n = 18). Similarly weak interaction was seen in HMS180 pups between age and frequency (multiple R^2^ = 0.0149, p = 0.8705, n = 18).

Age was a weak predictor of gamma frequency in both HMS15 pups (R^2^ = 0.1840, p = 0.0666, n = 19), and HMS180 pups (R^2^ = 0.0578, p = 0.3369, n = 18) (Fig. 4d). Correlations between weight and frequency were also nominal for both groups (HMS15: R^2^ = 0.0789, p = 0.2428, n = 19; HMS180: R^2^ =  = 0.0704, p = 0.2876, n = 18).

### Growth of PV+ interneuron population as predicted by weight

**Figure 5:**
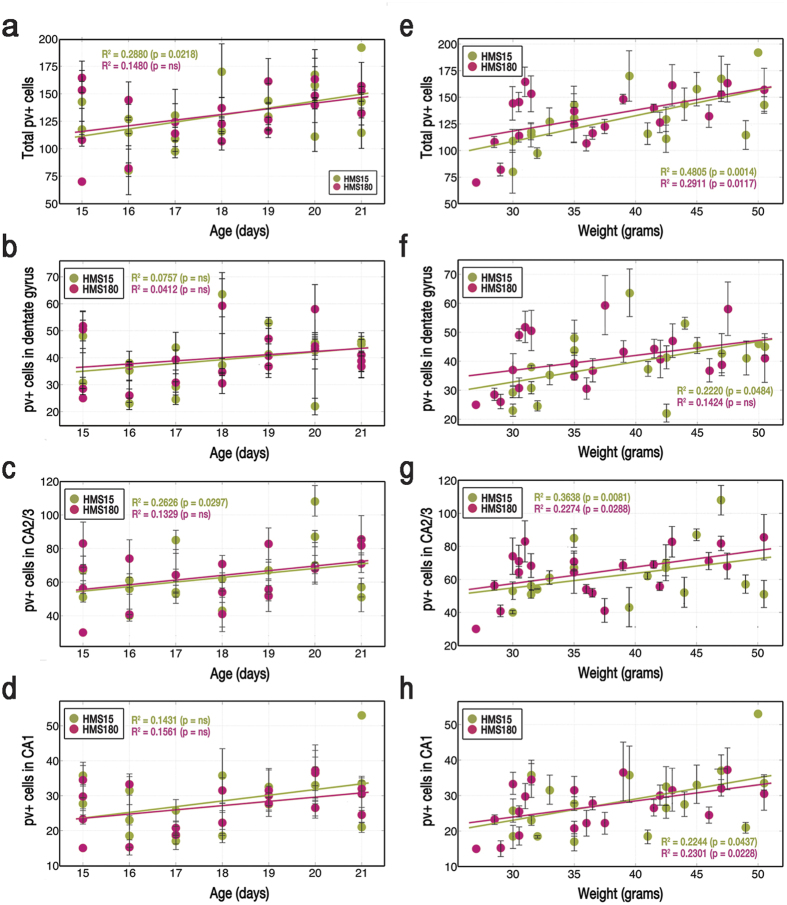
Effect of rearing condition (brief vs. extended maternal separations) on relationships between age (a–d) or weight (e–h) and number of PV+ cells in hippocampus. Cholinergic-receptor agonist carbachol (12.5 μM) was bath-applied to induce oscillations in 400 μM-thick slices, which were recorded from the pyramidal cell layer of CA3 in dorsal hippocampus. Gamma power was transformed by using the square root of gamma power in both figures and analyses, by which both were made linear. Where multiple sections from the same pup were analyzed, data points are averages of those sections. SEM bars indicate the standard error of the mean of sections taken from the same pup.

Correlations between age and the total number of parvalbumin-labeled cells per pup were moderate and significant for HMS15 pups (R^2^ = 0.2880, p = 0.0218, n = 18) but weak for HMS180 pups (R^2^ = 0.1480, p = 0.0846, n = 21) (Fig. 5a). Correlations in HMS groups between the number of parvalbumin-labeled cells and age also differed in CA2/3, with a moderate but significant correlation in HMS15 pups (R^2^ = 0.2626, p = 0.0297, n = 18) and nominal correlation in HMS180 pups (R^2^ = 0.1329, p = 0.1042, n = 21) (Fig. 5c). No significant correlations were observed in either of the groups between age and the number of parvalbumin-labeled cells in dentate gyrus (HMS15: R^2^ = 0.0757, p = 0.2692, n = 18; HMS180: R^2^ = 0.0412, p = 0.3777, n = 21) (Fig. 5b) or CA1 (HMS15: R^2^ = 0.1431, p = 0.1217, n = 18; HMS180: R^2^ = 0.1561, p = 0.0763, n = 21) (Fig. 5d).

Weight was a much stronger explanatory factor, predicting the total number of labeled cells (Fig. 5e: HMS15: R^2^ = 0.4805, p = 0.0053, n = 18; HMS180: R^2^ = 0.2911, p = 0.0117, n = 21), the number of stained cells in CA2/3 (Fig. 5g, HMS15: R^2^ = 0.3638, p = 0.0081, n = 18; HMS180: R^2^ = 0.2274, p = 0.0288, n = 21) and in CA1 (Fig. 5h, HMS15: R^2^ = 0.2244, p = 0.0437, n = 18; HMS180: R^2^ = 0.2301, p = 0.0228, n = 21) in a strong and significant manner for both HMS groups. In dentate gyrus, HMS15 pups showed a modest correlation between weight and number of parvalbumin-stained cells (R^2^ = 0.2220, p = 0.0484, n = 18), while HMS180 pups showed a weaker and non-significant correlation (R^2^ = 0.1424, p = 0.0917, n = 21) (Fig. 5f).

### Maturation of gamma as predicted by subfields’ PV+ populations

**Figure 6:**
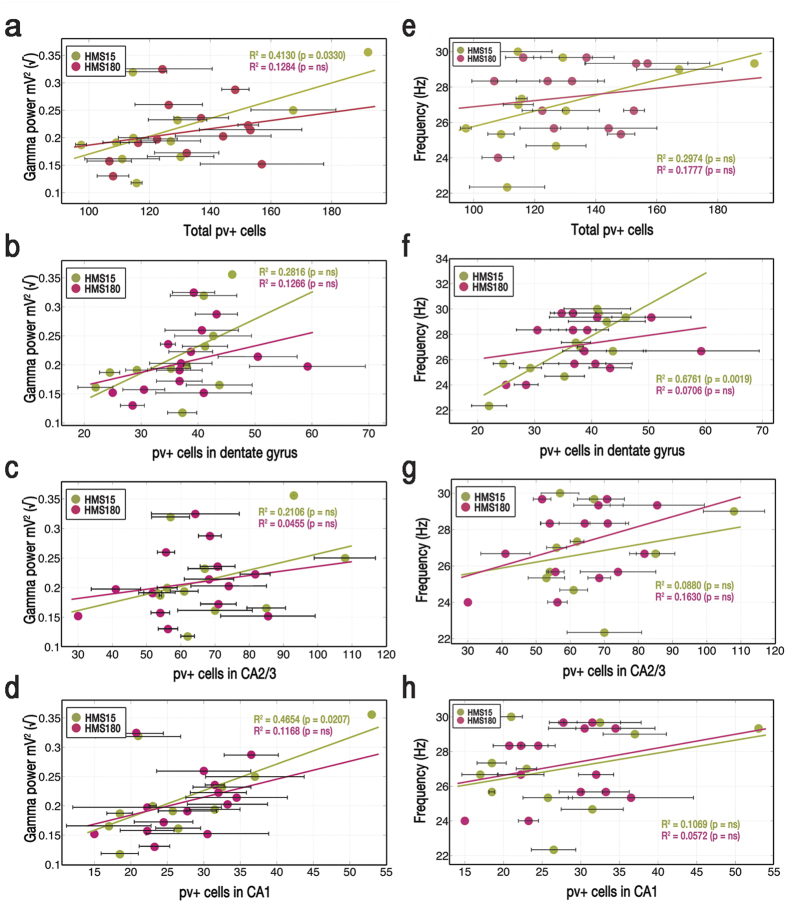
Subfield-specific effect of rearing condition (brief vs. extended maternal separations) on relationship between number of PV+ cells and gamma oscillation power (a–d) or frequency (e–h). Cholinergic-receptor agonist carbachol (12.5 μM) was bath-applied to acute hippocampal slices to induce gamma oscillations. The 20-minute interval spanning from 30 to 50 minutes after the start of recording (~20–25 minutes after introduction of drug) was used in all cases for data analyses. Where multiple sections from the same pup were analyzed, data points are averages of those sections. SEM bars indicate the standard error of the mean of sections taken from the same pup.

The number of PV+ cells in CA1 was a strong predictor of gamma power in HMS15 pups (R^2^ = 0.4654, p = 0.0207, n = 11) but not in HMS180 pups (R^2^ = 0.1168, p = 0.2317, n = 14) (Fig. 6d). A significant relationship was also seen in HMS15 pups between gamma power and total number of parvalbumin-stained cells per pup (R^2^ = 0.4130, p = 0.0330, n = 11; HMS180: R^2^ = 0.1284, p = 0.2084, n = 14) (Fig. 6a). No other strong relationships between gamma power and number of stained cells were observed. Correlations were non-significant in both groups in dentate gyrus (Fig. 6b, HMS15: R^2^ = 0.2816, p = 0.0931, n = 11; HMS180: R^2^ = 0.1266, p = 0.2118, n = 14) and CA2/3 (Fig. 6c, HMS15: R^2^ = 0.2106, p = 0.1557, n = 11; HMS180: R^2^ = 0.0455, p = 0.4639, n = 14).

The relationship between the total number of parvalbumin-stained cells per pup and gamma frequency was non-significant for both HMS groups (Fig. 6e, HMS15: R^2^ = 0.2974, p = 0.0830, n = 11; HMS180: R^2^ = 0.1777, p = 0.1334, n = 14). In HMS15 pups, however, the correlation between gamma frequency and number of immunostained cells specifically in dentate gyrus was very strong and highly significant (Fig. 6f, R^2^ = 0.6761, p = 0.0019, n = 11; HMS180 R^2^ = 0.0706, p = 0.3609, n = 14). The number of stained cells was not predictive of gamma frequency in either group in CA2/3 (Fig. 6g, HMS15: R^2^ = 0.0880, p = 0.3766, n = 11; HMS180: R^2^ = 0.1630, p = 0.1519, n = 14) or in CA1 (Fig. 6h, HMS15: R^2^ = 0.1069, p = 0.3255, n = 11; HMS180: R^2^ = 0.0572, p = 0.4120, n = 14; CA2/3: R^2^ = 0.1630, p = 0.1519, n = 14).

The relationship between age and weight was not differentially affected by HMS15 and HMS180 rearing (HMS15: R^2^ = 0.7273, n = 26, HMS180: R^2^ = 0.8517, n = 26, both p < 0.0001, figures not shown). No differences were noted in pups’ or dams’ appearance or behavior.

### HMS180 rearing affects temporal progress of electrophysiological maturation

Physiological development over time has been taken into account in our statistical analyses by looking at correlations of gamma power, gamma frequency and number of parvalbumin-immunopositive cells with age or weight. However, it is interesting to note that when described by simple averages of a seven-day period, very little difference was seen between the groups (Table 1). Pooling of the data by group across a seven day period, irregardless of age or weight, indicates that the duration of maternal separations, either 15 or 180 minutes, did not differentially affect the pups. Differences between the groups only emerge when the electrophysiological data is placed into context as a description of a developmental process, in conjunction with a critical variable representing progressive measures of maturity —herein represented by age or weight —and data points plotted at appropriately spaced intervals, i.e., 24 hours. Outside of this context, aberrance in the developing gamma oscillation network in HMS180 pups compared to the HMS15 pups, as shown by the preceding findings, is effectively masked.

## Discussion

Our findings indicate that neonatal maturation of hippocampal gamma oscillations is profoundly affected by chronic stress in the form of extended separations from the dam. Gamma oscillation power and frequency were significantly correlated with both age and weight in HMS15 pups but not in HMS180 pups. These strong correlations in HMS15 pups emerged only at later stages of maturation within the seven days of our study: at higher weights (>36 grams versus ≤36 grams) or later age (postnatal days 19 to 21 versus postnatal days 15 to 18); correlations were nominal in both groups prior to that, and in HMS180 pups remained so. This suggests that HMS180 rearing interferes with a critical phase of electrophysiological network maturation which is normally initiated during the third week of postnatal life.

PVBCs are last cells in the hippocampus to reach adult form and thus extremely vulnerable to the catabolic effects of premature exposure to glucocorticoids during the maternal separations phase of our study, PNDs 2 through 14; excessive exposure to glucocorticoids continues in HMS180 pups even after the stress hypo-response period closes. Developmental defects thus may rise at multiple levels, and HMS180 rearing may specifically affect gamma power and frequency in many ways. The complex arbor-like morphology of parvalbumin-expressing basket cells requires four to five weeks to complete, for example, as does the consequent electrophysiological maturation of the PVBC network. Aberrant calcium buffering at the axon terminal due to abnormalities in expression levels of parvalbumin would cause major changes in the firing and recovery properties of these cells, as would changes in passive membrane properties or synaptic connectivity as a function of anomalous cellular morphology. Relevant to the age of pups in our study as well is that increased oscillation frequency capability is in large part a function of changes in subunit expression both pre-and post-synaptically. The Kv3.2 subunit of the voltage-gated potassium channel, found predominantly on GABAergic fast-firing cells including parvalbumin-expressing interneurons^44^, facilitates recovery of sodium channels from inactivation and reduces the duration of after-hyperpolarization. By minimizing time in the refractory state, potassium channels having the Kv3.2 subunit facilitate high-frequency firing including gamma oscillations^45^. In hippocampus, Kv3.2 expression is seen predominantly in the pyramidal cell layer of CA1 and CA2/3 and at high levels in the hilar region of dentate gyrus^45^. Expression reaches adult levels by PND21^44^. In addition, the alpha-1 subunit of the GABA-A receptor is enriched at the postsynaptic side of synapses between parvalbumin-expressing basket cells, facilitating precisely synchronized activity in the basket cell network. The presence of this subunit in GABA-A receptors has been shown to facilitate faster activation and deactivation of those receptors in mature rats^46^. In immature rat hippocampus, alpha-1 subunits are present only in small number, but their expression increases greatly during the second and third weeks of postnatal life^47^. Increased expression of these subunits is also associated with developmental maturation of parvalbumin-expressing basket cell kinetics. Thus the irregular progression of gamma power expression seen in HMS180 pups may represent impairments of a variety of critical-period-dependent maturation processes.

Of the parvalbumin-immunopositive population seen in the HMS180s, cells that are not morphologically, neurochemically or electrophysiologically sound may not survive. This would align our findings with post-mortem studies that have found a deficit in parvalbumin-immunoreactivity in hippocampus and cortex in adults in the wake of schizophrenia, bipolar disorder or chronic stress^4^,^5^. Aberrant cells are more likely to atrophy and die off, or they may even change phenotype, as resulted from neonatal oxidative stress in a schizophrenia model, which in a recent study caused parvalbumin expression in GFP-labeled PVBCs to fall below detectable levels by or before the age of five weeks^48^. Similarly, work with post-mortem tissue from adult schizophrenic patients, in which PVBCs were identified by traits other than parvalbumin-immunoreactivity^49^, showed no difference from controls in the number of cells or density of GABAergic presynapatic PVBC axon terminals but did reveal significantly lower levels of parvalbumin mRNA. What has not been established with regard to schizophrenia or bipolar disorder is at what point in the lifetime these changes in PVBCs occur. Chronic early-life stress as modeled in this study is known to be a significant predictor of increased risk for both illnesses^1^,^2^,^3^,^50^,^51^,^52^.

Studies using the HMS180 protocol employed here have shown that HMS15-reared pups and adults are comparable in their behavior, cognitive abilities and neuroendocrine functions to naive animal facility-reared animals^53^,^54^. Results of our study show that during critical periods of neural development, up to and perhaps including the juvenile phase, factors which are potentially correlated with age and/or weight should be assessed before any degree of “homogeneity” in the group is assumed. Our findings suggest that the developmental trajectory of gamma oscillation power in naive pups is sufficiently steep between PNDs 15 and 21 that “pooling” their electrophysiological data could produce misleading results. This would be borne out not only by our findings but also by studies which have documented the electrophysiological and morphological maturation process of parvalbumin-expressing basket cells at time points between PNDs 6 and 25, illustrating the extraordinary transformation of these cells from “slow to fast signaling devices” within this very brief time^18^.

We conclude that HMS180 rearing, an animal model for the effects in humans of chronic early-life abuse, trauma or neglect, disrupts the developmental trajectory of the parvalbumin-expressing basket cell network and consequently impairs maturation of gamma oscillation activity in hippocampus. Impaired maturation of gamma oscillations may be a fundamental link between cognitive and memory deficits associated with chronic life stress and the etiologies of schizophrenia and bipolar disorder, which are thought to have bases in aberrant neurodevelopment, and for which those with histories of chronic early-life stress are at significantly increased risk.

## Additional Information

**How to cite this article**: Dricks, S. Effects of neonatal stress on gamma oscillations in hippocampus. *Sci. Rep.* **6**, 29007; doi: 10.1038/srep29007 (2016).

## Figures and Tables

**Figure 1:**
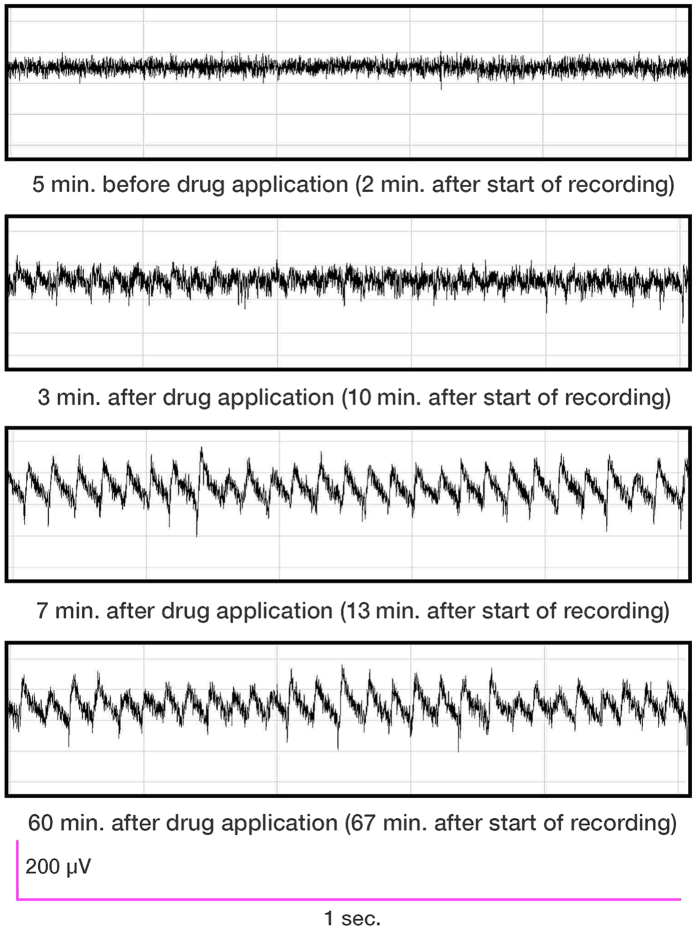
Local field potentials become synchronous and oscillatory following introduction of AchR agonist. Representative traces from an HMS15 pup on PND15 are shown before and after bath application of 12.5 μM carbachol. Baseline activity was recorded from acute slice for three minutes before drug was applied. Changes in local field potential were evident three minutes after introduction of the drug. Irregular oscillation-like fluctuations of the field potential gradually increased in temporal regularity and amplitude until a characteristic reverse-sawtooth pattern emerged. Oscillations continued for the duration of recording, shown here at 67 minutes.

**Figure 2:**
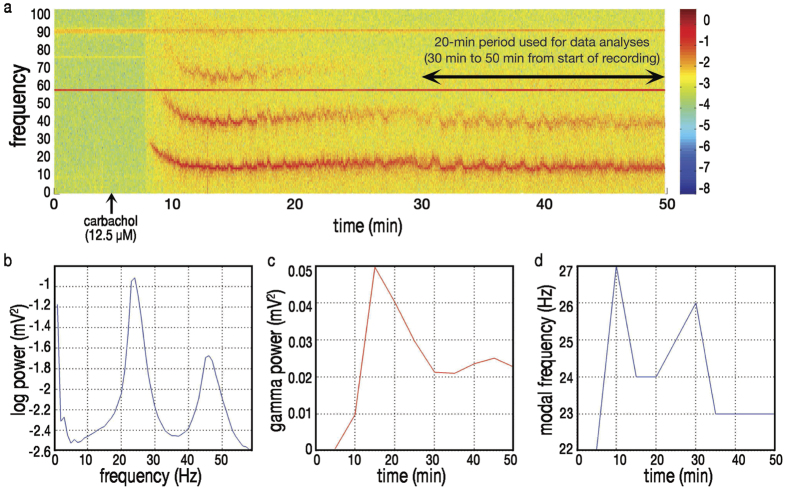
Changes in power and frequency of oscillatory activity over time. (**a**) Spectral density analysis from the first 50 minutes of the representative recording shown in Fig. 1 shows oscillation-like activity began within three minutes of bath application of carbachol (12.5 μM). As seen here, synchronous oscillations typically started at higher frequency than was seen for the duration of the recording: approximately four minutes after introduction of the drug, gamma-frequency oscillations appeared at 37 Hz and decreased rapidly to an average frequency of 24 Hz. Note first harmonic throughout (averaging 48 Hz) and second harmonic (averaging 72 Hz) at peak gamma power. In order to exclude from analyses harmonics of the gamma oscillations and 60 Hz “noise” from electrical mains, bandwidth was filtered offline, post-hoc, to a range of 20 to 40 Hz; all fundamental frequencies recorded were within this range. The 20-minute interval spanning from 30 to 50 minutes after the start of recording (~20–25 minutes after introduction of drug) was used in all cases for data analyses. (**b**) Oscillations were predominant in the range of 24 Hz, the fundamental frequency, with harmonic in the range of 48 Hz. (**c**) Gamma power of oscillations within the range of 20 to 40 Hz, averaged over non-overlapping five minute epochs. (**d**) Modal frequency of oscillations within the range of 20 to 40 Hz, averaged over non-overlapping five minute epochs.

**Figure 3:**
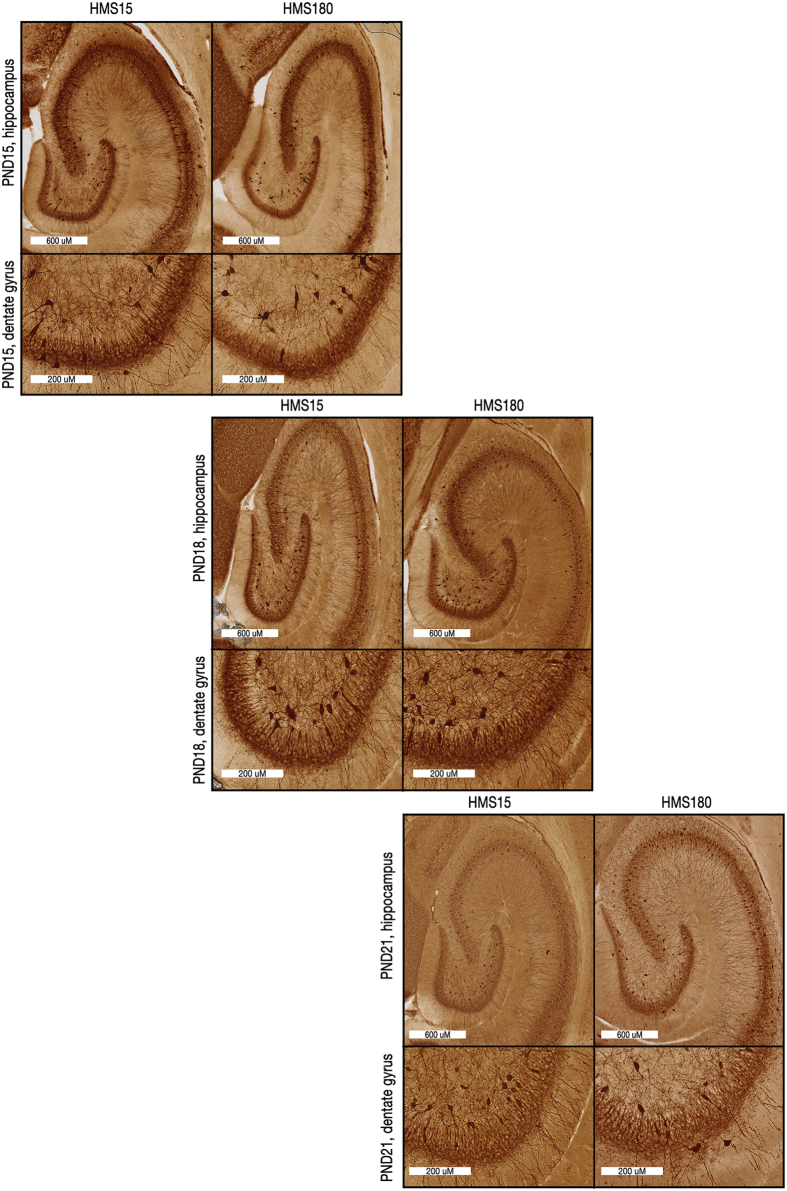
Maturation of PV+ interneuron network in dorsal hippocampus. 40 μM-thick sections from HMS15 and HMS180 pups at postnatal days 15, 18 and 21 were taken from the same longitudinal level of hippocampus as was used for electrophysiology. Image of hippocampus proper and the accompanying image of detail from dentate gyrus in each set are from the same pup. Staining of cell bodies was consistently prominent within and proximal to the pyramidal and granule cell layers and in the hilus. Stained cell bodies averaged ~15–20 μM in diameter at the widest part, and within the cell layer were typically oriented in parallel to principal cells, as determined by soma shape and site of emergence of processes from the soma. Density of PV-immunopositive basket cell axon terminals synapsing on unlabeled principal cell bodies identifies principal cell soma within the layer.

**Table 1:** Effects of rearing condition (brief vs extended maternal separations) on 7-day averages of somatic and electrophysiological maturation.

Collective averages of PNDs 15–21, per HMS group	HMS15	HMS180
Gamma oscillation activity:		
Mean frequency (Hz)	27.26	27.54
Mean gamma power (mV^2^)	0.0414	0.0412
Mean weight (grams)	37.78	36.56
Min weight (grams)	26	27
Max weight (grams)	50	50.5
Number of immunopositive cells in all subfields, mean	131.24	131.84
Number of immunopositive cells in all subfields, mean SEM	9.12	10.42
Mean number of immunopositive cells, CA1	28.8	26.79
Mean number of immunopositive cells, CA2/3	63.16	64.59
Mean number of immunopositive cells, dentate gyrus	39.42	40.23

Data pooled from PNDs 15 to 21 did not distinguish groups by rearing condition in averages of gamma power, gamma frequency, body weight or number of PV+ cells per subfield in hippocampus. SEM is the standard error of the mean for all sections from the same pup; “mean SEM” is the mean of all SEMs for each HMS group.
